# Enhanced Analgesic Effects and Gastrointestinal Safety of a Novel, Hydrogen Sulfide-Releasing Anti-Inflammatory Drug (ATB-352): A Role for Endogenous Cannabinoids

**DOI:** 10.1089/ars.2019.7884

**Published:** 2020-10-08

**Authors:** Soraia K.P.F. Costa, Marcelo N. Muscara, Thibault Allain, Jorge Dallazen, Larissa Gonzaga, Andre G. Buret, David J. Vaughan, Christopher J. Fowler, Gilberto de Nucci, John L. Wallace

**Affiliations:** ^1^ Department of Pharmacology, Institute of Biomedical Sciences, University of Sao Paulo, São Paulo, Brazil.; ^2^ Department of Biological Sciences, University of Calgary, Alberta, Canada.; ^3^ Antibe Therapeutics, Inc., Toronto, Canada.; ^4^ Department of Pharmacology and Clinical Neuroscience, Umeå University, Umeå, Sweden.; ^5^ Department of Pharmacology, University of Campinas, Campinas, Brazil.; ^6^ Department of Physiology and Pharmacology, University of Calgary, Calgary, Canada.

**Keywords:** inflammation, pain, hydrogen sulfide, ulcer, analgesic, ketoprofen, cannabinoid

## Abstract

***Aims:*** The covalent linking of nonsteroidal anti-inflammatory drugs to a hydrogen sulfide (H_2_S)-releasing moiety has been shown to dramatically reduce gastrointestinal (GI) damage and bleeding, as well as increase anti-inflammatory and analgesic potency. We have tested the hypothesis that an H_2_S-releasing derivative of ketoprofen (ATB-352) would exhibit enhanced efficacy without significant GI damage in a mouse model of allodynia/hyperalgesia.

***Results:*** ATB-352 was significantly more potent and effective as an analgesic than ketoprofen and did not elicit GI damage. Pretreatment with an antagonist of the CB1 cannabinoid receptor (AM251) significantly reduced the analgesic effects of ATB-352. The CB1 antagonist exacerbated GI damage when coadministered with ketoprofen, but GI damage was not induced by the combination of ATB-352 and the CB1 antagonist. *In vitro*, ATB-352 was substantially more potent than ketoprofen as an inhibitor of fatty acid amide hydrolase, consistent with a contribution of endogenous cannabinoids to the analgesic effects of this drug. Blood anandamide levels were significantly depressed by ketoprofen, but remained unchanged after treatment with ATB-352.

***Innovation:*** Ketoprofen is a potent analgesic, but its clinical use, even in the short term, is significantly limited by its propensity to cause significant ulceration and bleeding in the GI tract. Covalently linking an H_2_S-releasing moiety to ketoprofen profoundly reduces the GI toxicity of the drug, while boosting analgesic effectiveness.

***Conclusion:*** This study demonstrates a marked enhancement of the potency and effectiveness of ATB-352, an H_2_S-releasing derivative of ketoprofen, in part, through the involvement of the endogenous cannabinoid system. This may have significant advantages for the control and management of pain, such as in a postoperative setting.

## Introduction

Postoperative pain relief facilitates recovery from injury (12, 15), but optimal perioperative or postoperative treatments remain problematic. While opioids are very effective analgesics, the significant risk of addiction is a major barrier to their use, even in the short term. Nonsteroidal anti-inflammatory drugs (NSAIDs) can be effective in managing pain, but their use is limited by the substantial risk of bleeding, particularly in the gastrointestinal (GI) tract (24). While proton pump inhibitors and histamine H2-receptor antagonists can reduce such bleeding in the stomach and proximal duodenum, they offer no benefit in the remainder of the GI tract, where significant NSAID-induced ulceration and bleeding can occur (25a). Indeed, suppressors of acid secretion can significantly exacerbate ulceration and bleeding in the intestine (17, 30, 35, 36).

In animal models, derivatives of NSAIDs that contain a hydrogen sulfide (H_2_S)-releasing moiety have been shown to produce markedly lower levels of GI damage and bleeding than the parent NSAIDs (8, 27). These H_2_S-NSAIDs produce more potent suppression of prostaglandin synthesis (cyclooxygenase activity; COX) than that observed with the parent NSAID, particularly in humans (26). A marked increase in GI safety of an H_2_S-releasing NSAID was recently demonstrated in humans (29). Rates of upper GI ulceration were examined endoscopically in a double-blind, phase 2 clinical trial involving 244 subjects taking equieffective doses of either naproxen or ATB-346 for two weeks (29). The rate of ulceration in the subjects taking naproxen was 42.2%, while that in subjects taking ATB-346 was dramatically reduced (to 2.5%; *p* < 0.001).

**Innovation**

Recent studies have provided evidence that covalently linking a hydrogen sulfide (H_2_S)-releasing moiety to a nonsteroidal anti-inflammatory drug dramatically reduces gastrointestinal (GI) toxicity and increases therapeutic potency. This study demonstrates (using a novel H_2_S-releasing derivative of ketoprofen) that the enhanced GI safety and increased efficacy/potency are mediated, in part, *via* the anandamide–cannabinoid system. The data are consistent with the hypothesis that slow release of H_2_S can substantially reduce hemorrhagic damage in the GI tract despite marked suppression of cyclooxygenase activity.

In the present study, we evaluated the analgesic efficacy of an H_2_S-releasing derivative of ketoprofen (ATB-352) *versus* equimolar doses of ketoprofen in a mouse model of enhanced sensitivity to pain (mechanical allodynia/hyperalgesia). Ketoprofen is a very potent NSAID, but is among the most GI toxic of this class of drugs (7, 34). ATB-352 has been shown to be GI safe in animal models despite profound suppression of COX activity (8) and it does not activate mu opioid receptors at concentrations up to 30 μg/mL. We also examined the potential contribution of the endogenous cannabinoid, anandamide, to the analgesic potency and GI safety of ATB-352.

## Results

### ATB-352 exhibits enhanced analgesic potency *versus* ketoprofen

**FIG. 1.**
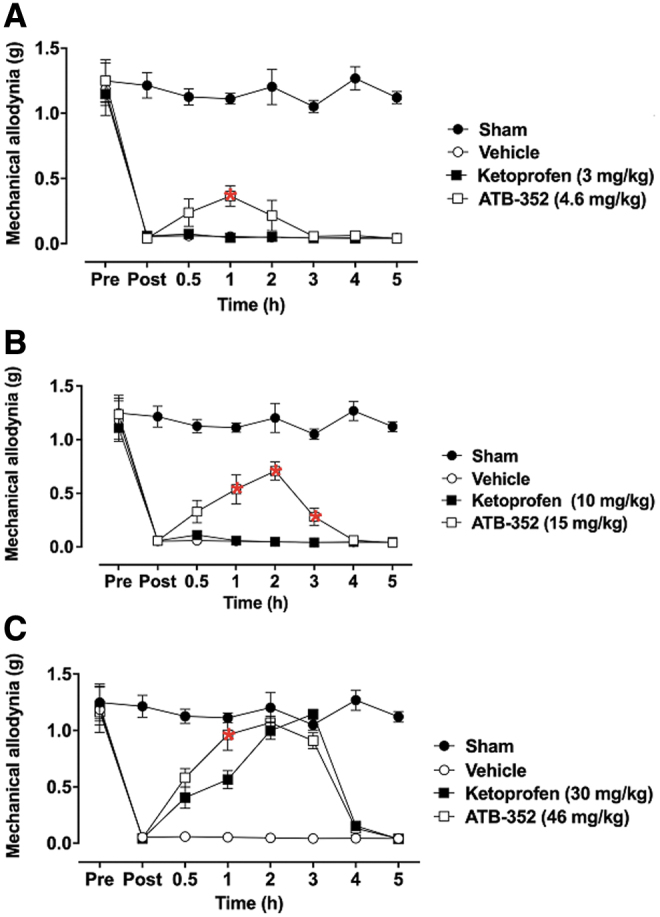
**Enhanced analgesic effects of ATB-352 *versus* ketoprofen on mechanical postoperative allodynia in mice.** The presence of mechanical allodynia is indicated by a decrease in the threshold force necessary for the animal response (*i.e.*, paw withdrawal). Mice were orally pretreated with ketoprofen (3, 10, or 30 mg/kg); (**A–C**), respectively; *n* = 6, equimolar doses of ATB-352 (4.6, 15, or 46 mg/kg; *n* = 6), or vehicle (1 mL/kg; *n* = 6). The control group (sham) did not receive any treatment (*n* = 6). Data are expressed as mean ± SEM changes in paw withdrawal force thresholds measured before (pre) and 24 h after the incision (post) and then at 0.5, 1, 2, 3, 4, and 5 h after treatment. *Red asterisks* indicate a significant difference between the mice treated with ATB-352 and the corresponding ketoprofen-treated group (*p* < 0.05). Data were analyzed by one-way ANOVA, followed by the Bonferroni multiple comparison test.

In vehicle-treated mice, there was a rapid and marked increase in mechanical allodynia 24 h after the incision had been made (Fig. 1). This remained unchanged throughout the 5-h postincision observation period (Fig. 1A–C). The mechanical allodynia responses were not affected by the lowest or intermediate doses of ketoprofen (3 and 10 mg/kg), but treatment with equimolar doses of ATB-352 (4.6 and 15 mg/kg) significantly attenuated postoperative pain from 1 to 3 h postdose (Fig. 1A, B). A higher dose of ketoprofen (30 mg/kg; p.o.) was required to significantly reduce pain sensitivity (Fig. 1C), and at an equimolar dose (46 mg/kg), ATB-352 markedly reduced the pain response in mice 0.5, 1, 2, and 3 h after treatment (Fig. 1C).

### Role of CB1 in pain response and GI injury

**FIG. 2.**
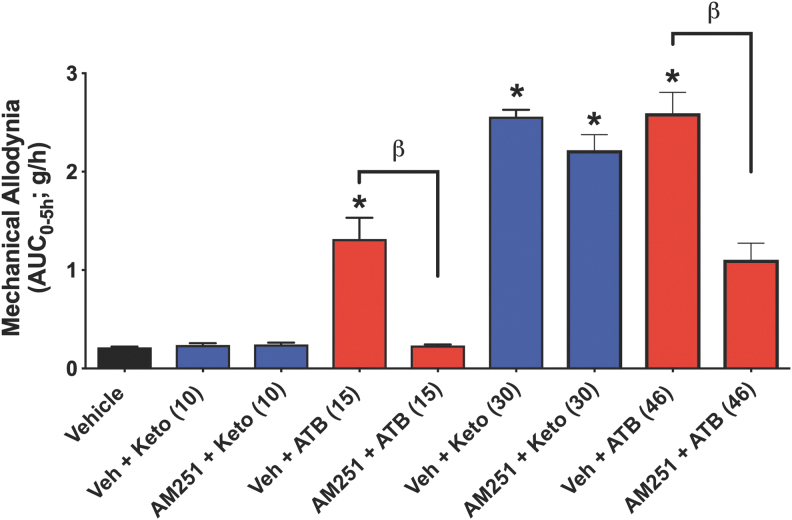
**Reduction of pain in the mechanical allodynia model in mice (area under the curve of a 5-h observation period) by ketoprofen *versus* ATB-352 and effects of pretreatment with a cannabinoid antagonist (AM251; 3 mg/kg i.p.).** At a dose of 10 mg/kg, ketoprofen was not effective in reducing pain, in contrast to significant allodynia with an equimolar dose of ATB-352 (15 mg/kg; *p < 0.05 *vs.* the vehicle-treated group). At a higher dose (30 mg/kg), ketoprofen did produce a significant reduction of mechanical allodynia, comparable with that observed with an equimolar dose of ATB-352 (46 mg/kg). Pretreatment of ketoprofen-treated mice with AM251, a cannabinoid (CB1) antagonist, did not affect the extent of mechanical allodynia. In contrast, the mechanical allodynia observed in mice treated with equimolar doses of ATB-352 was significantly reduced by pretreatment with the CB1 antagonist (^β^*p* < 0.05). Data were analyzed by one-way ANOVA, followed by the Bonferroni multiple comparison test, and data are presented as the mean ± SEM (*n* = 6 per group).

Anandamide, an endogenous cannabinoid, has been implicated as an analgesic mediator through activation of CB1 receptors as well as contributing to NSAID-related analgesia (22). The analgesic effects of ketoprofen at 30 mg/kg were comparable with those of one-third the molar equivalent dose of ATB-352. Pretreatment with the CB1 antagonist, AM251 (22), reversed the analgesic effect of ATB-352, but did not reduce the analgesic effect of ketoprofen (Fig. 2). This suggests that a significant component of the analgesic effects of ATB-352 is attributable to endogenous cannabinoids.

**FIG. 3.**
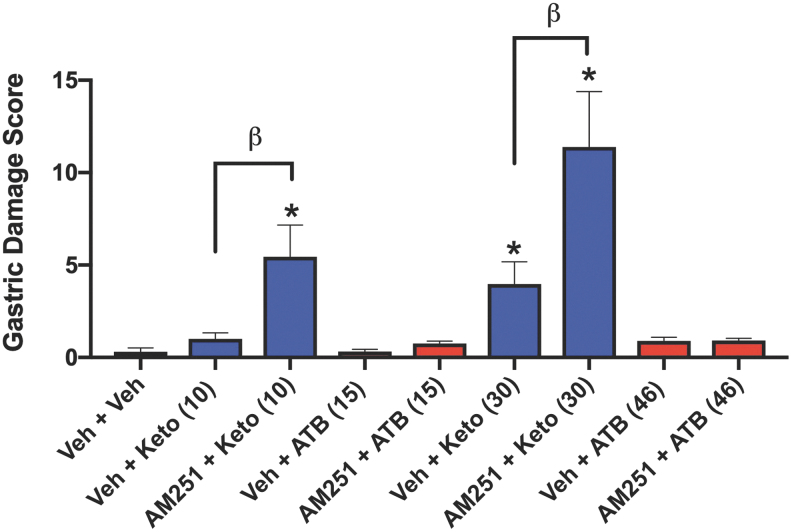
**Induction of gastric mucosal hemorrhagic injury by ketoprofen, but not by ATB-352, and a potential role for endogenous cannabinoids in protecting the gastrointestinal tract.** Groups of six mice each were treated orally with vehicle, ketoprofen (10 or 30 mg/kg), or equimolar doses of ATB-352, and gastric damage was blindly quantified 5 h later (measurement of areas of hemorrhagic erosions, with histological confirmation). The effects of oral pretreatment with a cannabinoid (CB1) receptor antagonist, AM251 (30 min before ketoprofen or ATB-352), were also examined. Ketoprofen administration caused dose-dependent statistically significant (*p < 0.05) levels of gastric mucosal damage. Pretreatment with the CB1 antagonist markedly increased the severity of ketoprofen-induced gastric damage (^β^*p* < 0.05). In contrast, ATB-352 caused negligible gastric mucosal damage, even when the mice were pretreated with the CB1 antagonist. Data were analyzed by one-way ANOVA, followed by the Bonferroni multiple comparison test, and data are presented as the mean ± SEM (*n* = 6 per group).

Anandamide can also increase the resistance of the GI mucosa to injury, as demonstrated in several animal models (8, 21, 22). We have previously reported that antagonism of CB1, but not CB2, receptors reversed the protective effects of a cannabis extract against naproxen-induced GI damage (28). Moreover, activation of CB1 (but not CB2) has been shown to enhance colitis-associated pain in rats (20). In the present study, pretreatment with the selective CB1 receptor antagonist, AM251, resulted in an approximately threefold increase (*p* < 0.05) in the extent of gastric damage in mice treated with 30 mg/kg ketoprofen, while in mice treated with an equimolar dose of ATB-352 plus the CB1 antagonist, there was negligible GI damage (Fig. 3).

**FIG. 4.**
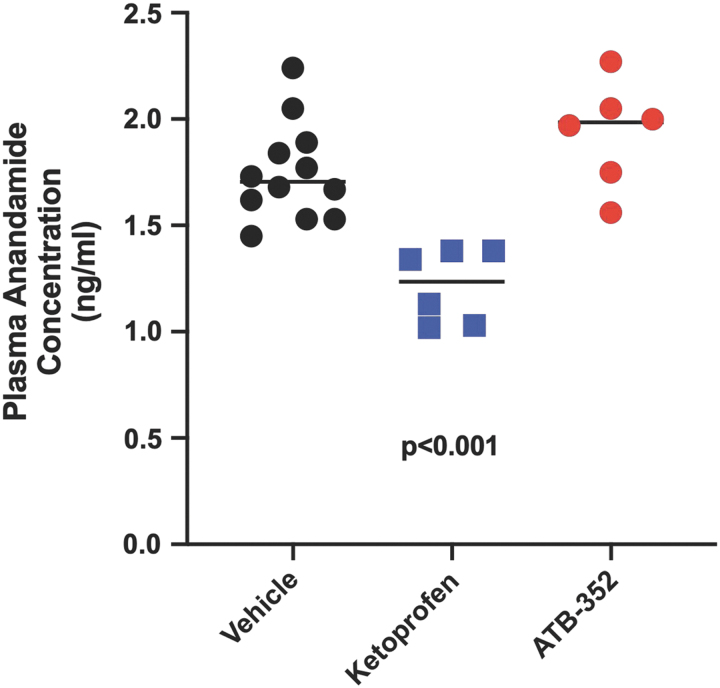
**Levels of anandamide in blood were significantly reduced (*p* < 0.001) in mice treated with ketoprofen (10 mg/kg) 5 h before sampling, in sharp contrast to the lack of effect of an equimolar dose of ATB-352.** Data were analyzed by one-way ANOVA, followed by the Bonferroni multiple comparison test, and data are presented as the mean ± SEM (*n* = 6 per group).

These data on pain relief and reduction of GI damage are consistent with changes in blood levels of anandamide following administration of ketoprofen or ATB-352. Levels of anandamide in blood taken from mice 5 h after administration of 10 mg/kg ketoprofen were markedly reduced compared with control levels (*p* < 0.001), while anandamide levels in mice treated with an equimolar dose of ATB-352 were unchanged (Fig. 4).

### *In vitro* inhibition of fatty acid amide hydrolase

**FIG. 5.**
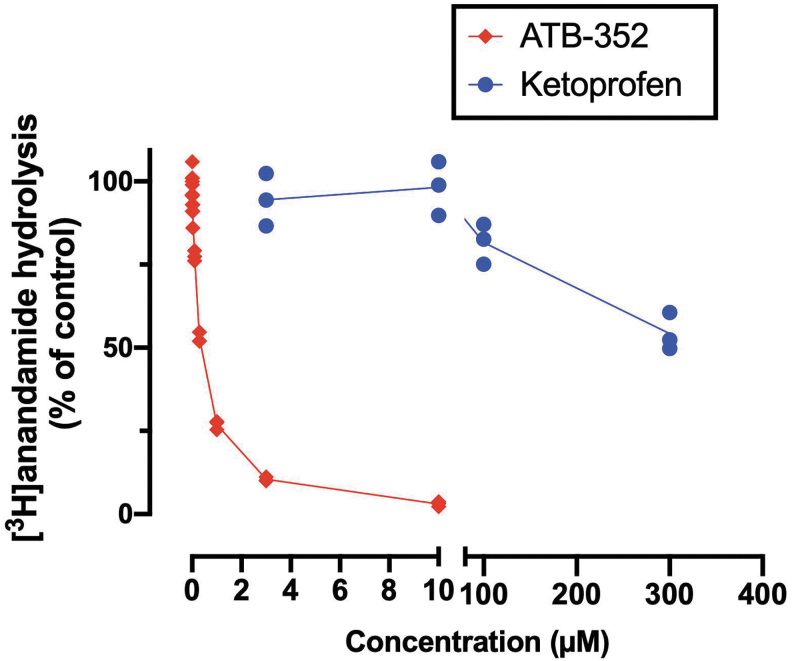
**Inhibition of FAAH by ketoprofen and ATB-352 (an H_2_S-releasing ketoprofen derivative).** Data are shown as individual values from three experiments. The potency of ATB-352 was substantially greater than that of ketoprofen. p*I*_50_ values for ATB-352 and ketoprofen were 6.46 ± 0.02 and <2.52, respectively, corresponding to mean IC_50_ values of 340 n*M* and >300 m*M*. Data are shown as the means (*n* = 3 per group). FAAH, fatty acid amide hydrolase; H_2_S, hydrogen sulfide.

COX-2 inhibitors can inhibit fatty acid amide hydrolase (FAAH), thereby reducing catabolism of anandamide and contributing to analgesia and GI protection (13). We compared the activity of ATB-352 *versus* ketoprofen for inhibition of FAAH activity *in vitro*, as described previously (13). As shown in Figure 5, the inhibition curve for ATB-352 was shifted significantly to the left compared with the inhibition curve for ketoprofen. This is consistent with the markedly increased potency of ATB-352 *versus* ketoprofen that was observed in our *in vivo* studies. A significant leftward shift of the inhibition curve was also observed when ATB-346 was compared with naproxen in this *in vitro* assay (unpublished), although not so profound as with ATB-352/ketoprofen.

## Discussion

In recent years, the biological importance of H_2_S in many organs and systems has become increasingly clear. Extensive studies have documented the many beneficial anti-inflammatory effects of this gaseous mediator and its role in promoting GI resistance to injury, as well as resolution of inflammation and tissue damage (6, 18, 25, 32). Several groups have developed novel H_2_S-releasing drugs in the hope that they will be useful for treatment of a range of diseases (1, 20, 37). Our work has focused on the design of novel anti-inflammatory and analgesic drugs, including ATB-346, which was recently shown to be substantially more GI safe than the parent drug (naproxen) in a phase 2 clinical trial. Naproxen is among the most widely used anti-inflammatory drugs, but its use is associated with a high incidence of GI bleeding and ulceration (23). Thus, in a GI endoscopy study, 2 weeks of treatment with naproxen led to the development of upper GI ulcers in 42.2% of subjects (29). This was in sharp contrast to the ulcer incidence of only 2.5% of subjects (*p* < 0.001) treated with an equieffective dose of ATB-346 (28).

Some of the earliest animal and human studies of ATB-346 suggested that the drug was a substantially more potent inhibitor of COX activity than the parent drug. Indeed, in phase 1 clinical trials, we observed that ATB-346 was approximately six times more potent than naproxen and produced COX inhibition that persisted twice as long as that achieved with naproxen (31). Laboratory animal studies of the ketoprofen derivative, ATB-352, yielded similar findings as with ATB-346; that is, more potent inhibition of COX than with equimolar doses of ketoprofen and a substantial reduction of GI damage (8, 11). Ketoprofen is among the most potent and GI-damaging NSAIDs (34) and is used for treatment of a range of inflammatory conditions (*e.g.*, arthritis and gout) as well as for managing postsurgical pain. We undertook the present study with a goal of gaining a better understanding of the mechanisms underlying the increased potency and GI safety of ATB-352 *versus* ketoprofen. Since ATB-352 does not activate mu opiate receptors at concentrations up to 30 μ*M* (unpublished data), this drug may represent an attractive alternative to opioids for severe pain relief and therefore a potential solution to at least part of the opioid crisis. In the present study, it exhibited a significant increase in potency of pain relief in a well-characterized surgical incision model (19) compared with ketoprofen (steady-state plasma ketoprofen levels in the ATB-352-treated animals were ∼3–5 μ*M*). Moreover, in contrast to generation of hemorrhagic GI lesions in mice treated with ketoprofen, such damage was not observed in the mice treated with ATB-352.

**FIG. 6.**
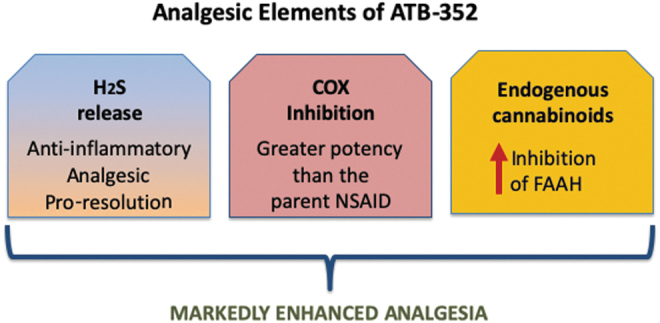
**The analgesic effects of ATB-352 include contributions from H_2_S, cyclooxygenase inhibition, and endogenous cannabinoids.** H_2_S-releasing NSAIDs have consistently exhibited substantially enhanced potency *versus* the parent drug. H_2_S-releasing NSAIDs (and H_2_S donors) markedly reduce the severity of NSAID-induced gastrointestinal damage while also promoting resolution of inflammation and repair of tissue damage. H_2_S-releasing NSAIDs also exhibit markedly enhanced potency with respect to inhibition of FAAH, resulting in maintained or enhanced levels of endogenous cannabinoids, such as anandamide. As shown in this study, endogenous cannabinoids contribute significantly to the enhanced analgesic effects of ATB-352 as well as to the markedly improved gastrointestinal safety *versus* a conventional NSAID (ketoprofen). NSAID, nonsteroidal anti-inflammatory drugs.

In recent years, substantial evidence has been generated for a significant interplay between arachidonic acid metabolism and endogenous cannabinoids. FAAH catalyzes the hydrolysis of anandamide and other similar lipid amides (22). COX-2 inhibitors can reduce this hydrolysis, leading to increased levels of endogenous cannabinoids (including anandamide) and thereby enhancing analgesia. In contrast, treatment with a CB1 antagonist can inhibit the actions of endogenous cannabinoids, thereby reducing analgesia. Thus, in the present study, oral administration of a selective CB1 antagonist (AM251) profoundly reduced the analgesic effects of ATB-352, while not affecting those of ketoprofen. On the other hand, increased FAAH-catalyzed hydrolysis of endogenous cannabinoids can reduce analgesia and increase susceptibility to GI ulceration (9, 21, 22, 28). This was confirmed in the present study by the observation that treatment with the selective CB1 antagonist resulted in a profound increase in hemorrhagic damage in the GI tract of the ketoprofen-treated mice (5-to 10-fold *vs.* the ATB-352-treated mice). The lack of damage in the ATB-352-treated mice following administration of the CB1 antagonist strongly suggests the existence of additional protective mechanisms, likely related to H_2_S (Fig. 6). Plasma anandamide levels decreased significantly in the ketoprofen-treated mice, measured 5 h after drug administration, which was consistent with the observed increase in susceptibility to GI injury. In contrast, anandamide levels remained unchanged in the mice treated with ATB-352. These observations are consistent with the study by Goodman *et al.* (10), who reported that NSAIDs that inhibit both COX and FAAH exhibit decreased GI damage, possibly as a result of increased levels of fatty acid ethanolamides.

The marked increase in FAAH inhibitory potency of ATB-352 over that of ketoprofen was confirmed in an *in vitro* assay system. The concentration–response curve for ATB-352 was shifted dramatically to the left compared with ketoprofen. An increase in potency in the FAAH assay was also observed for ATB-346, the H_2_S-releasing naproxen derivative (unpublished).

There is a large body of evidence regarding the ability of H_2_S to reduce inflammation and pain. Cenac *et al.* (3) demonstrated enhanced nociceptive effects of an H_2_S-releasing derivative of trimebutine and Distrutti *et al.* (4) demonstrated a similar dose-dependent phenomenon with an H_2_S-releasing derivative of mesalamine, in both cases, using colonic distention-induced pain as an endpoint. We have similarly examined distention-induced pain responses in rats using cardiovascular endpoints, and once again, there was a significantly greater reduction of pain with the H_2_S-releasing derivative than with the parent drug (26). This is consistent with studies we have performed using this assay to evaluate an H_2_S-releasing derivative of naproxen (ATB-346), suggesting an important contribution of H_2_S to analgesic effects of ATB-352 and other such compounds (3, 14, 26). Kida *et al.* reported that H_2_S reduced microglial activation and central nervous system inflammation (14). Inhaled H_2_S reduced upregulation of inflammatory cytokines and prevented the associated neuropathic pain behavior (14). They speculated that inhaled H_2_S prevented neuropathic pain through inhibition of activation of microglia in the spinal cord. Lucarini *et al.* have extensively studied isothiocyanates, which are natural or synthetic compounds that exhibit antihyperalgesic and anti-inflammatory effects (16). Importantly, the authors reported that there was a strict association between the release of H_2_S and pharmacodynamic observations.

Consistent with laboratory-based studies of ATB-352, Antibe Therapeutics' lead compound (ATB-346) has been shown to be a much more potent inhibitor of COX than the parent drug in humans, with significant reduction of osteoarthritis-associated pain (in an open-label study) (31). H_2_S release from ATB-346 was confirmed in the recent phase 2 endoscopy trial by Antibe Therapeutics, Inc. (Toronto, ON, Canada). On average, plasma H_2_S levels increased by ∼50% in subjects receiving ATB-346 *versus* subjects receiving naproxen. In addition to beneficial effects that may be conferred by the release of H_2_S, the present study provides evidence of a significant contribution of endogenous cannabinoids, including anandamide, to both the enhanced efficacy and GI safety of ATB-352.

## Materials and Methods

### *In vivo* studies

Adult, male BALB/c mice (20–30 g; 7–8 week old), obtained from the animal rearing facilities at the Faculty of Medicine, University of São Paulo, Brazil, were used in the experiments in accordance with the Ethical Principles for Animal Research established by the ICB/USP Ethics Committees for Animal Use in Research, according to the National Council for Animal Experimentation Control principles, consistent with the Animal Welfare Act. According to the internal laboratory rules, euthanasia was to be performed if severe distress related to the protocol developed during the experiment. The mice were group-housed in a temperature-controlled room at 22°C with a 12-h light/12-h dark cycle and allowed free access to food (Nuvilab CR-1; Quimtia S/A, Brazil) and filtered water.

ATB-352 was provided by Antibe Therapeutics, Inc., while ketoprofen was purchased from Sigma-Aldrich (Oakville, ON, Canada). Suspensions were prepared in 0.5% carboxymethylcellulose (CMC; Cromoline Química Fina Ltd., Diadema, SP, Brazil) with the aid of a vortex mixer.

The mice were orally treated with vehicle (4 mL/kg of 0.5% CMC), ketoprofen (3, 10, or 30 mg/kg), or equimolar doses of ATB-352 (4.6, 15, or 46 mg/kg).

### Reduction of postoperative pain

The ability of ATB-352 and equimolar doses of ketoprofen to reduce postoperative pain was examined using a plantar incision model, as described by Brennan *et al.* (2), and modified for use in mice by Pogatzki and Raja (19). The mice were anesthetized with inhaled 1.5%–2% isoflurane (v/v in O_2_), delivered *via* a plastic nose cone, followed by antiseptic (10% povidone–iodine solution) preparation of the right hind paw. Subsequently, a 5-mm longitudinal incision was made with a number 11 surgical blade, starting 2 mm from the proximal edge of the heel and extending toward the toes, through the plantar foot skin and fascia. With the aid of a curved forceps, the underlying muscle and tendons were carefully elevated, then replaced to the normal anatomical position. After controlling bleeding, the skin was sutured with 6–0 nylon in the middle of the incision. The wound was coated with 10% povidone–iodine solution and mice were allowed to recover in their cages until the following day. Control (sham-treated) mice underwent anesthesia and antiseptic procedures, without an incision. At the end of the experiments, all mice were euthanized with an overdose of isoflurane, followed by cervical dislocation.

Measurement of mechanical paw withdrawal threshold was assessed blindly using von Frey filament stimulation (0.04–4 g; North Coast Medical, Inc., Morgan Hill, CA) perpendicularly to the ventral surface of the right hind paw to determine the 50% threshold (g) based on the up and down paradigm (5). Groups of 6 mice each were randomly assigned to be treated orally with vehicle (0.5% carboxymethylcellulose, 4 mL/kg), ketoprofen (3, 10, or 30 mg/kg), or equimolar doses of ATB-352 (4.6, 15, or 46 mg/kg). The mice were then placed individually into a transparent plastic box (18 × 11 × 20 cm; Insight, Inc., Ribeirão Preto, SP, Brazil) on an elevated mesh platform and allowed to acclimatize for 1–2 h before testing. In a blinded manner, von Frey filaments ranging from 0.4 to 4.0 g were applied to the plantar surface of the right hind paw (avoiding the toes, heel, and pads) for 1–2 s with an interstimulus interval of at least 5 s. The mechanical allodynia (g) was evaluated immediately before the incision (basal response), at 24 h after surgery (time 0), and at 0.5, 1, 2, 3, 4, and 5 h thereafter. At the end of the experiments, all mice were euthanized with an overdose of isoflurane, followed by cervical dislocation.

### Plasma anandamide levels and effects of CB1 antagonist

After completion of the assessment of pain, blood was drawn from mice for measurement of plasma anandamide levels (*i.e.*, 5 h after drug/vehicle administration; ketoprofen at 10 mg/kg and ATB-352 at 15 mg/kg). The mice were anesthetized with inhaled 2% isoflurane (v/v in O_2_) to facilitate blood collection using a heparinized syringe. Plasma was obtained from blood samples by centrifugation (2000 *g* at 4°C) for 10 min and then frozen on dry ice and stored at −80°C. Plasma aliquots (50 μL) were diluted (1:2) in phosphate-buffered saline (pH 7.4) and centrifuged (10,000 *g* at 4°C) for 15 min. The anandamide levels were quantified using a competitive inhibition enzyme-linked immunosorbent assay, according to the manufacturer's instructions (Biomatik Corporation, Cambridge, ON, Canada). The results were interpolated with an anandamide standard curve and expressed as ng/mL of plasma.

The potential roles of CB1 in the analgesic and GI-damaging effects of ketoprofen and ATB-352 were also examined. Groups of mice (*n* = 6) were orally administered vehicle (phosphate-buffered saline) or the CB1 antagonist, AM251 (3 mg/kg i.p.) (22), and 30 min later, they were orally treated with ketoprofen (10 or 30 mg/kg) or equimolar doses of ATB-352 (15 or 46 mg/kg). Postoperative pain responses were blindly recorded 1–5 h after administration of the test drugs, as above. At the end of the experiment, the gastric and duodenal mucosae were blindly scored (macroscopically and microscopically) for hemorrhagic damage, as described previously (30).

## Abbreviations Used

ANOVA: analysis of variance
CB: cannabinoid
COX: cyclooxygenase
FAAH: fatty acid amide hydrolase
GI: gastrointestinal
H_2_: histamine receptor-2
H_2_S: hydrogen sulfide
NSAID: nonsteroidal anti-inflammatory drug
